# Reaching the World Health Organization elimination targets for schistosomiasis: the importance of a One Health perspective

**DOI:** 10.1098/rstb.2022.0274

**Published:** 2023-10-09

**Authors:** Adriana V. Díaz, Martin Walker, Joanne P. Webster

**Affiliations:** ^1^ Department of Pathobiology and Population Sciences, Royal Veterinary College, Hatfield AL9 7TA, UK; ^2^ Department of Infectious Disease Epidemiology, London Centre for Neglected Tropical Disease Research, Faculty of Medicine, Imperial College, London W2 1PG, UK

**Keywords:** One Health, schistosomiasis, elimination, livestock, transmission

## Abstract

The past three years has seen the launch of a new World Health Organization (WHO) neglected tropical diseases (NTDs) roadmap, together with revised control and elimination guidelines. Across all, there is now a clear emphasis on the need to incorporate a One Health approach, recognizing the critical links between human and animal health and the environment. Schistosomiasis, caused by *Schistosoma* spp. trematodes, is a NTD of global medical and veterinary importance, with over 220 million people and untold millions of livestock currently infected. Its burden remains extremely high in certain regions, particularly within sub-Saharan Africa, despite over two decades of mass preventive chemotherapy (mass drug administration), predominantly to school-aged children. In Africa, in contrast to Asia, any zoonotic component of schistosomiasis transmission and its implications for disease control has, until recently, been largely ignored. Here, we review recent epidemiological, clinical, molecular, and modelling work across both Asia and Africa. We outline the evolutionary history and transmission dynamics of *Schistosoma* species, and emphasize the emerging risk raised by both wildlife reservoirs and viable hybridization between human and animal schistosomes. To achieve the 2030 WHO roadmap elimination targets, a truly multi-disciplinary One Health perspective must be implemented.

This article is part of the theme issue ‘Challenges and opportunities in the fight against neglected tropical diseases: a decade from the London Declaration on NTDs’.

## 1. Introduction

Momentum in the fight against neglected tropical diseases (NTDs), 20 epidemiologically complex conditions that predominantly affect impoverished communities [1], has been building alongside a change in targets from morbidity control to elimination. With renewed emphasis on the need to incorporate a One Health approach, ‘an integrated, unifying approach that aims to sustainably balance and optimize the health of people, animals and ecosystems' [2, p. viii], the World Health Organization (WHO) has launched a series of documents over the past 3 years, including a revised 2021–2030 NTDs roadmap [3], a revised *Guideline on control and elimination of human schistosomiasis* [4], a companion One Health approach document for action against NTDs [2], a global strategy on water, sanitation and hygiene (WASH) to combat NTDs [5], and a 2022–2026 *One Health joint plan of action* in conjunction with other international agencies [6]. These documents serve to embed One Health at the heart of control efforts for NTDs with significant environmental or zoonotic epidemiological characteristics and stress the importance of One Health in sustainably improving the intertwined health of humans, animals and their ecosystems [2].

Schistosomiasis is among the most important NTDs and is estimated to infect a quarter of a billion people worldwide [7,8]. This debilitating parasitic disease is present in 78 tropical and subtropical countries, with major foci across Asia, Africa and South America [3], and a potentially emerging focus in southern Europe [9]. However, its burden is placed overwhelmingly on sub-Saharan Africa (SSA), where 90% of human schistosome infections occur [7,10,11], causing approximately 24 000 deaths and at least 2.5 million disability-adjusted life years (DALYs) [3]. The parasite's environmental infective stages—snail-infective miracidia and mammal-infective cercariae—are restricted to freshwater habitats of the snail intermediate host, thus schistosomiasis is considered a water-borne disease [5], with ongoing debates for and against its classification as a vector-borne disease [12,13]. Importantly, the clinical consequences of infection by schistosomes are experienced not only by humans, but also by non-human mammalian definitive hosts [14], many of which can act as reservoirs of subsequent human infection for certain schistosome species [15–17].

As of January 2020, of 78 endemic countries 51 are targeted for mass preventive chemotherapy (i.e. mass drug administration, MDA, using praziquantel) by WHO owing to moderate to high schistosome transmission [3]. The 2030 WHO schistosomiasis elimination goals call for its elimination as a public health problem (EPHP) from all endemic countries—42 in Africa, ten in the Americas, three in Southeast Asia, six in the western Pacific, 16 in the eastern Mediterranean and one in Europe [18]—with EPHP defined as less than 1% heavy-intensity infections (≥50 eggs per 10 ml urine or ≥400 eggs per gram of stool are indicative of a heavy-intensity infection [18]). After 20 years of large-scale MDA, morbidity profiles have shifted; thus a revised EPHP definition based on overall infection prevalence instead of heavy-intensity infections has recently been advocated [19]. In addition to EPHP, interruption of transmission (IoT) in 25 of the 78 endemic countries has also been set as a WHO schistosomiasis elimination goal, with IoT defined as absence of infection in humans [3]. WHO's EPHP goal has been met in China since 2015 [20] but further national control efforts aim for transmission control as well as transmission interruption. Transmission control is defined by Chinese authorities as less than 1% infection prevalence in humans and livestock, plus two consecutive days of negative malacological surveys and no acute schistosomiasis cases [20]—although schistosomiasis is most commonly a chronic disease, an acute presentation has been described in humans [10] and livestock [21]. Transmission interruption is defined by Chinese authorities as absence of infection in humans, livestock and snails for five consecutive years [20].

The WHO roadmap [3] identifies a number of actions required to reach the schistosomiasis elimination targets, which include: more comprehensive MDA strategies (e.g. expanding to include more demographic groups, ensuring access to therapeutics, developing alternatives to praziquantel); snail control (e.g. developing appropriate guidelines for targeted snail control and alternative control methods); improved diagnostics—for humans and animals—for micro-mapping; and the creation of cross-sectoral mechanisms to coordinate key sectors like WASH, environmental management and animal health. The roadmap also expounds on the risk posed by zoonotic reservoirs in Asia and beyond, advocating wider use of veterinary public health interventions [3]. Specific points for consideration include improved sanitation and management of animal waste, keeping animals away from transmission sites and treating animals with praziquantel. The companion One Health approach WHO document [2, p. xi] ‘provides guidance on the One Health actions needed by major stakeholders and how to support a paradigm shift towards One Health in national NTD programmes', and is complemented by the *One Health joint plan of action* [6] framework to support and expand One Health capacities at global, regional, and national level. Hence, the One Health approach is now firmly embedded within WHO guidance on eliminating schistosomiasis.

Operationalizing One Health measures for zoonotic NTDs is nevertheless challenging (see [22]). For instance, despite long-standing knowledge of the effectiveness of anti-rabies dog vaccination in preventing human deaths [23], capacity building for mass dog vaccination is still a critical action required to reach the 2030 rabies elimination targets [3]. Moreover, the recent detection of *Dracunculus medinensis* infections in animals [24] has triggered a focus on emerging animal reservoirs to reach dracunculiasis eradication targets [3]. Indeed, there are now more infections recorded in non-human mammals (mostly domestic dogs, some domestic cats and a few baboons [25]) than in humans [24]. Interestingly, a non-classical transmission pathway in dogs likely contributing to disease persistence has been identified in Chad [25].

In this perspective, we first consider recent advancements in understanding the evolutionary history and transmission dynamics of *Schistosoma* spp., before outlining the emerging risk posed by wildlife and livestock reservoirs of infection and hybridization between human-infective and livestock-infective schistosomes. We then highlight the importance of adopting a One Health approach to eliminate schistosomiasis and discuss potential interventions targeting livestock in SSA that could facilitate progress towards elimination. Finally, we discuss the challenges and next steps associated with implementation of a One Health strategy.

### (a) Natural history of *Schistosoma* spp.

The natural history of schistosomes has been marked by their expanding geographical range, host-switching and diversification. Modern molecular biology and genomics indicate an Asian origin of schistosomes, disfavouring the traditional African-origin hypothesis [14]. The prevailing ‘out of Asia’ theory detailed by Lawton *et al.* [14] posits that schistosomes evolved as parasites of rodents from an avian schistosomatid ancestor. The *Schistosoma japonicum* clade, which still retains distinct genomic similarities with avian parasites, is thought to be the antecedent of the genus [14]. Further speciation likely followed an additional host shift to ungulates that concurred with the diversification of their molluscan intermediate hosts [14]. Over 40 vertebrate species can serve as hosts for *S. japonicum* [10,15,26,27]*,* a generalist strategy that may have helped preserve ancestral genomic features [14]. All three *S. japonicum* clade species responsible for human disease in Asia—*S. japonicum, Schistosoma mekongi* and *Schistosoma malayensis*—are zoonotic (i.e. transmitted between animals and humans) [26].

Schistosomes likely invaded Africa on at least two separate occasions [14]. The first occurrence gave rise to the *Schistosoma hippopotami* clade, including two species described from hippos. A second ancestral invasion during the radiation of mammals into Africa during the mid-Miocene caused a population bottleneck that formed a proto-*Schistosoma mansoni* clade. This ultimately gave rise to two distinct clades of African schistosomes, the *S. mansoni* clade and the *Schistosoma haematobium* clade [14]. The more ancestral *S. mansoni* clade comprises two species that parasitize rodents, *Schistosoma rodhaini* and *S. mansoni* [28]. The latter species is the predominant cause of human intestinal schistosomiasis in SSA and also infects non-human primates [10,29–34] (for a review on non-human primate *S. mansoni* reservoirs in Africa see [35]), although it is often classed as an exclusively human parasite [36]. The Atlantic slave trade is thought responsible for the geographical expansion of *S. mansoni* to the Caribbean and South America [37,38]. The more recent *S. haematobium* clade comprises nine species, including the human-infective species *S. haematobium*, *Schistosoma intercalatum* and *Schistosoma guineensis*, and the species of veterinary health importance causing ruminant intestinal schistosomiasis, *Schistosoma bovi*s, *Schistosoma curassoni* and *Schistosoma mattheei*, with the remaining species, *Schistosoma margrebowiei*, *Schistosoma leiperi* and *Schistosoma kisumuensis,* mostly found in wildlife [39–42]. The postulated reinvasion of Asia from Africa by the *Schistosoma indicum* clade, formed of three species of veterinary significance, did not involve a host switch but the continued parasitization of ungulates and rodents [14].

Despite the prevailing perception of schistosomiasis as a predominantly human disease [10,36], the *Schistosoma* genus has shown a remarkable ability to diverge and adapt to new hosts and habitats that has persisted beyond its evolutionary origins. This is evident not only among members of the Asian *S. japonicum* clade, but also among several species of both African clades, as exemplified by the establishment and persistence of *S. mansoni* in the Caribbean [38,43,44], and the recent expansion of *S. haematobium* **×** *S. bovis* hybrids into Europe ([45] but see [9]). This adaptability presents increasingly recognized challenges to the control and elimination of schistosomiasis and epitomizes the necessity for a holistic One Health approach.

### (b) Hybridization between *Schistosoma* species

Concerns about *Schistosoma* hybridization were initially raised by phenotypic observations of unusual egg and worm morphologies deemed to present intermediate characteristics of different species [21,39,46,47], as well as changes in cercarial emergence and shedding patterns [48]. Subsequently, enzymatic studies [39,49–51], and crucially, molecular techniques, have uncovered strong evidence of the interspecific hybridization of schistosomes [28,39,52–55]. Interbreeding between different species can result in the creation of hybrid offspring (hybridization) or, if backcrossing of an interspecific hybrid with a parent species occurs, in the introduction of single chromosomal regions or genes from one species to the other (introgressive hybridization, also known as introgression) [33,56,57]. Such processes can drive genetic variation, with the potential to alter the morbidity, host range, and drug susceptibility of parasites [33,53,57].

Phylogenetically close schistosome species with overlapping endemicity are more amenable to hybridization. Nonetheless, whilst cases of natural hybridization between *S. japonicum* clade species have not been found [39], instances of likely non-viable hybridization between distantly related *S. mansoni* with *S. haematobium* have been recorded in humans in Mali [58], Cameroon [59], Senegal [60], Côte d'Ivoire [61] and Kenya [62]. Although, *S. mansoni* clade hybrids have been found in snails in Tanzania [63] and Kenya [28], most evidence of schistosome hybridization involves species within the *S. haematobium* clade, with such interspecific hybrids having been recorded across SSA (table 1; for a review on *Schistosoma* hybridizations see [39]).

**Table 1.** Hybrids between species of the *Schistosoma haematobium* clade recently reported across sub-Saharan Africa.

reported *S. haematobium* clade interspecific hybrid combination^a^	host infected by the schistosome hybrid	type of sample evaluated for molecular evidence	country	reference^b^
*S. bovis* × *S. haematobium*	human	miracidia	Benin	[48]
*S. haematobium* × *S. bovis*	rodent	miracidia, plus snail infection	Benin	[64]
*S. bovis* × *S. haematobium*	ruminant (cattle)	miracidia, plus snail infection	Benin	[48]
*S. haematobium* × *S. bovis*; and *S. bovis* × *S. haematobium*	human	miracidia	Cameroon	[65]
*S. guineensis* × *S. haematobium*; and *S. haematobium* × *S. bovis*	human/snail	sequencing of F1 male worms (field samples used in rodent laboratory infection)	Cameroon	[55]
*S. haematobium* × *S. bovis*	human	miracidia	Côte d'Ivoire	[66]
*S. bovis* × *S. haematobium*	snail	cercariae	Côte d'Ivoire	[67]
*S. haematobium* × *S. mattheei*; and *S. haematobium* × *S. bovis*	human	atypical eggs	Malawi	[68]
*S. haematobium* × *S. bovis*	human	atypical eggs from travellers	Mali	[69]
*S. haematobium* × *S. bovis*	human/snail	sequencing of F1 male worms (field samples used in rodent laboratory infection)	Mali	[55]
*S. bovis* × *S. curassoni*; and *S. bovis* × *S. haematobium* × *S. curassoni*	human	miracidia	Niger	[47]
*S. haematobium* × *S. bovis*; and *S. haematobium* × *S. bovis* × *S. curassoni*	snail	cercariae	Niger	[70]
*S. haematobium* × *S. bovis*	human	miracidia	Nigeria	[71]
*S. haematobium* × *S. bovis*	human/snail	sequencing of F1 male worms (field samples used in rodent laboratory infection)	Nigeria	[55]
*S. haematobium* × *S. bovis*	human	miracidia	Senegal	[72]
*S. bovis* × *S. haematobium*	human	miracidia	Senegal	[16]
*S. haematobium* × *S. bovis*	snail	cercariae	Senegal	[16]
*S. haematobium* × *S. bovis*	rodent	female worm	Senegal	[73]
*S. bovis* × *S. curassoni*	ruminant (cattle, sheep, goat)	miracidia	Senegal	[16]
*S. mattheei* × *S. haematobium*	human	sera from travellers (acute cases), followed by sequencing	South Africa	[74]

^a^Hybrid combination as reported in reference.

^b^Reported since Leger & Webster [39].

The interspecific mixing of *S. haematobium* with *S. bovis* has been highlighted in several West African foci [55,75] (table 1). Within different areas of the Senegal River basin, a schistosomiasis hotspot, a heterogeneous distribution of *S. haematobium* **×** *S. bovis* hybrids has been reported [72], and a positive association between the frequency of such hybrids and the prevalence of *S. mansoni* has been identified (i.e. higher risk of hybrid infection in villages with high *S. mansoni* prevalence) [72]. A possible explanation for this association is the increased susceptibility of humans to livestock-infective schistosomes following pure or mixed *S. mansoni* infection [72]. Active immune response suppression caused by *S. mansoni* is hypothesized to facilitate infection with *S. bovis* or F1 hybrids, consequently driving hybrid transmission [72]. The observed geographical heterogeneity in *S. haematobium* × *S. bovis* hybrid distribution may be explained by: water contact behaviour and immunology of the host; compatibility of hybrids with the locally predominant snail species; more intensive livestock rearing around the shores of Lac de Guiers (where most hybrids have been found); and ongoing selection of *S. bovis* introgressed genes due to their possible adaptive benefits [72].

Interestingly, since 2013, a hybrid *S. haematobium* **×** *S. bovis* isolate likely originating from West Africa seems to have adapted successfully to the local snail and human population in Corsica, France [76]. This hybrid strain has caused several outbreaks of human schistosomiasis in Corsica without an apparent need for animal reservoirs [76,77]. Overwintering of the Corsica hybrid schistosome strain within the local snail host has been recently demonstrated, providing a potential explanation of local persistence in this newly established focus [78]. Previous single-species infection studies in baboons produced *S. mattheei* eggs but no *S. bovis* eggs [79] nor *S. curassoni* eggs [80], suggesting the latter two livestock-specific schistosomes may require heterospecific mixing to produce viable offspring in humans [47]. However, recent evidence of ‘pure’ *S. bovis* viable human infection in Nigeria [71] has proven that this species can in fact develop fully as a single species in people. Furthermore, contrary to prevailing thought on the epidemiology of hybridizations between livestock- and human-associated *S.* *haematobium* clade schistosomes, recent studies carried out in West Africa have demonstrated that human infection by *S. bovis* hybrids is common (table 1).

Genome-wide restriction-site-associated DNA sequencing (RADseq) has shown an increased fitness and viability (so-called hybrid vigour) of *S. haematobium* clade hybrids, which may at least partially explain the change in endemic landscape over a period of 25 years from predominantly intestinal (caused by *S. guineensis*) to urogenital human cases in sympatric regions of Central Africa [55]. Furthermore, recent genomic studies have found *S. haematobium* clade hybridizations to be both ancient [81] and ongoing [82]. Both hybrid vigour and the rise in interspecific schistosome encounters, facilitated by the increased movement of people and animals, will likely increase the frequency of hybridizations as interventions are intensified to meet elimination goals [57].

### (c) Importance of a One Health approach

Several schistosome species are currently widely recognized as zoonotic, particularly livestock-infective species of the *S. japonicum* clade in Asia. In Africa, livestock-infective species of the *S. haematobium* clade (i.e. *S. bovi*s, *S. curassoni* and *S. mattheei­*) which tend to hybridize with *S. haematobium* can also be classified as zoonotic, though the significance of domestic ruminants as reservoirs for human transmission is yet to be clarified [75,83]. Land use change, climate change, and expanding trade, migration and mobility will likely increase parasite hybridization as well as zoonotic spillover [82]. The introduction of schistosomes into new habitats can be mediated by the livestock trade (e.g. the recent discovery of *S. bovis* in Zanzibar [84]), economic migration and displacement and indeed tourism (e.g. in 2017 a cluster of acute cases was reported in Belgian travellers returning from South Africa and associated with *S. mattheei* and *S. haematobium* hybridization [74]). The increased migration of animals and/or humans in conjunction with man-made ecological change (e.g. dam construction, irrigation systems, agriculture and deforestation) will continue altering schistosomes' geographical range, further complicating control efforts [2].

In China, where sustained control efforts (including livestock interventions) have successfully eliminated *S. japonicum* from many endemic foci, the risk of resurgence from both domestic and wildlife animals remains an ongoing challenge (e.g. in 2014, the infection prevalence documented in rodents, goats and dogs was above 9% in Hunan province [85]). Elsewhere, the finding in 2019 of a green monkey infected with *S. mansoni* in St Kitts—where schistosomiasis was thought to have been eliminated in the 1970s—raises several questions, including reintroduction of the parasite to the island, or the existence of a sylvatic cycle maintained by non-human primates [43].

The role of wild rodents as reservoirs of zoonotic schistosomes is being progressively uncovered, with rodents found to be infected with *S. mansoni* in the Caribbean [44,86], Brazil [87] and Africa [73,88,89]. Moreover, in Africa, where the majority of intestinal (caused by *S. mansoni*) and urogenital (caused by *S. haematobium*) human schistosomiasis cases occur, wild rodents have been confirmed to be naturally infected with *S. bovis* and S. *haematobium* clade hybrids [64,73]. Importantly, infected livestock are also relevant sources of zoonotic spillover, particularly, cattle infected with *S. bovis* [16,17].

Approximately 12 million people have been estimated to be at risk of zoonotic schistosomiasis in Asia [90]. Such estimates are yet to be produced for Africa. Resolving the nature of multi-host transmission at a local level remains challenging [91], though recent modelling work [15,17,57] highlights the emerging risk of zoonotic hybrids and other zoonotic schistosomes in Africa [83] and questions whether the traditional top-down, human-centric approach to control and elimination is suitable [92]. Increased understanding of the population structure of schistosomes and their response to selective pressures induced by MDA requires interdisciplinary work, including a combination of molecular, epidemiological and modelling analyses [93]. Given the noteworthy high prevalence of *S. bovis* genes in parasites recently recovered from Nigerian children [71], there is a pressing need to more completely understand the population structure and transmission dynamics of schistosomes infecting African livestock [94] and their spatio-temporal distribution, particularly where zoonotic hybrid prevalence is highest [72]. Similarly, the role of rodents, non-human primates and other relevant animal species warrants further research.

Improved understanding of the eco-epidemiology of schistosomiasis at the human–animal interface and the economic and sociological factors affecting the feasibility and effectiveness of control interventions has been recently proposed as policy pillars for the sustainable elimination of zoonotic schistosomiasis [90]. Zoonotic *S. japonicum* clade and *S. haematobium* clade schistosomes, as well as species that cause animal schistosomiasis, ought to be considered when setting animal health and veterinary public health goals to accompany existing public health elimination goals [3]. Furthermore, the genital pathology of other *S. haematobium* clade species besides *S. haematobium* in urogenital schistosomiasis cases in women and men must be elucidated, including the risk of genital forms of disease from zoonotic hybrids.

### (d) Targeting livestock schistosomiasis in sub-Saharan Africa

Interventions to tackle the emerging risk of zoonotic hybrids in Africa include preventing susceptible livestock from coming into contact with contaminated water bodies (e.g. by providing drinking water in troughs, contamination of communal water with livestock faeces could be minimized [95]), treatment of infected livestock and limiting interaction with wildlife [83]. Reliance on other proposed interventions such as replacement of livestock (e.g. for agricultural management of wetlands) [83] may not be advisable, given schistosomes' tendency for host-switching and range of potential animal reservoirs. For instance, in the case of *S. japonicum*, buffaloes, cattle and horses are considered main reservoirs in parts of Indonesia [96], whereas transmission between dogs and humans and between rodents and humans has been reported, respectively, in areas of the Philippines [97] and China [98].

A key unresolved question for the establishment of a comprehensive One Health strategy is whether extending praziquantel treatment to relevant animal reservoirs in SSA would be epidemiologically and economically beneficial [34]. For example, in 2022, a study assessing the financial impact of livestock schistosomiasis on subsistence farmers of northern Senegal indicated that the impact of withholding interventions substantially surpassed the cost of testing and treatment [99]. Such findings indicate that there is a significant economic burden placed on traditional farmers by this disease, and thus favour, from an economic standpoint, the extension of praziquantel treatment to livestock in SSA.

The use of praziquantel for livestock schistosomiasis is already thought to be widespread in parts of SSA [34,99], but misuse is reported widely, with farmers often employing subtherapeutic doses, inappropriate products instead of suitable veterinary formulations, and incomplete treatment regimens. Thus, while there exists demand for livestock schistosomiasis control, there is a need for a structured approach [100]. Initial exploration of a herd-level test-and-treat (TnT) approach for cattle using a mathematical transmission model suggests that up to 75% of bovine case-years in highly endemic settings and up to 85% of bovine case-years in lower transmission contexts could be averted [100]. The potential impact of TnT interventions could thus be substantial. There remain several outstanding challenges before TnT interventions could be implemented, including the availability of sensitive and specific point-of-care diagnostics [3,75] (see [101] for a systematic review and meta-analysis of diagnostics for non-human schistosomiasis) and suitable veterinary formulations [100]. Moreover, a TnT approach can only partially mitigate the risk of promoting drug resistance, an ominous spectre given the exclusive reliance on praziquantel for the treatment of human and animal schistosomiasis (table 2). Nonetheless, the clear contribution of cattle to spillover infections in humans and subsequent enhanced risks of hybridization, in addition to the operational feasibility of such an intervention in livestock (compared with wild animals), plus the economic benefits for impoverished communities, justify development of a comprehensive control strategy focused on domestic ruminants [100].

**Table 2.** Concepts and considerations regarding mitigating the risk of emerging resistance to praziquantel.

anthelmintic resistance	Anthelmintic resistance is a heritable change that allows the survival of parasites when exposed to previously efficacious doses of chemotherapy [102]. Emergence of resistance occurs as the selective advantage of drug-resistant strains progressively surpasses that of the wild-type and is established if the resistant phenotype remains able to be transmitted between hosts [102]. Over-reliance on a single anthelmintic drug (praziquantel) and current lack of pharmaceutical alternatives for the treatment of schistosomiasis [103] increases the vulnerability of control and elimination programmes to emerging resistance. There remains no conclusive evidence for the emergence of praziquantel resistance in natural schistosome populations, although instances of reduced efficacy (e.g. for *S. mansoni* human infection in Uganda [104]) and unsatisfactory clinical improvement (e.g. for *S. haematobium* clade human infection caused by interspecific hybrids in Senegal [105]) have been reported.
veterinary use of anthelmintics	The use of anthelmintics in the veterinary field is thought to increase the risk of emerging drug resistance [34], owing in part to the unstructured way in which drugs are administered in some settings. A structured test-and-treat approach [100] has potential to partly counteract such risks, although the risk of emerging resistance can never be completely avoided [102]. Notwithstanding, even after decades of extensive praziquantel use in livestock in China, no evidence of drug resistance under field conditions has been reported to date [102].
evolutionary pressure of interventions and presence of zoonotic hybrids	A bidirectional effect between the evolutionary pressure of control interventions and the presence of zoonotic hybrids is probable [57], with potential impacts including reduced praziquantel efficacy and changing morbidity profiles [57], as recently reported in people infected with *S. haematobium* × *S. bovis* hybrids in Senegal [105], in addition to a potentially heightened role of animals in transmission as prevalence in humans decreases [15,57].
laboratory schistosomes response to selective pressures	Laboratory studies (see [102]) have produced changes in schistosome fecundity, virulence, drug susceptibility, infectivity, and population genetics [106–114] within a few generations and using a range of selective pressures. How such experimental laboratory studies translate to natural populations remains unclear.
refugia of drug-susceptible genes	Untreated zoonotic reservoirs may act as refugia—a pool of drug-susceptible genes that can dilute resistant genes—which slow the emergence of drug resistance [33,34]. Refugia arise within untreated individuals in the at-risk populations and other animal species relevant to the parasite's life cycle, and perhaps even immature schistosomes that are not susceptible to praziquantel [102]. The size and relevance of refugia will vary depending on the transmission dynamics of the schistosome species. For example, since *S. japonicum* has several vertebrate hosts that can act as refugia, it is proposed that there exists a low likelihood of emerging resistance [102].
emerging drug resistance	Mass drug administration can exert a sufficiently strong population-level selective pressure for the evolution of resistant strains [102], with repeated chemotherapy predicted to exert selective pressures that favour parasite resistance alleles [115]. The emergence of resistance, especially when it is a recessive trait, tends to follow a sigmoid-type temporal dynamic and therefore is often identified only during or after the exponential-type growth phase that follows an initial slow rise [102]. This underlines the importance of early detection to give time for mitigating interventions to be implemented. Relaxation of constraining density-dependent processes (e.g. on parasite fecundity) after chemotherapy can enhance the spread of drug resistance, depending on the particular density-dependent process at play [115].

The efficacy of praziquantel against livestock schistosomiasis—cattle and small ruminants (sheep and goats)—is a long-standing pending research need that must be answered if a One Health treatment approach is to be safely implemented, with additional knowledge gaps including ruminant species-specific praziquantel pharmacokinetics and pharmacodynamics, drug bioavailability and ideal administration route. Commercial veterinary formulations for the treatment of livestock schistosomiasis with praziquantel are not currently widely available in SSA [34]. Hence, enhanced and alternative supply lines of praziquantel for veterinary usage will also require development to meet the increased demand.

Current estimates of the prevalence and geographical extent of livestock schistosomiasis are incomplete [16] and require a comprehensive epidemiological mapping approach to redress [34]. Crucial information at a local level on domestic ruminants’ exposure to contaminated water sources will further prospects for the design and implementation of strategies that complement treatment by mitigating re-infection. Continuous surveillance and monitoring to detect early signs of emerging resistance in both humans and animals are also paramount to safeguard praziquantel. Such activities would include monitoring prevalence, clinical outcomes and drug efficacy (e.g. egg reduction rates), assessment of temporospatial genetic diversity, population structure and gene flow, and measurement of allele frequency changes mediated by treatment [102] (table 2).

Currently, there are no commercially available schistosomiasis vaccines for either humans or animals [26,83], although this is an active area of research and development (for recent reviews see [116] and [117]), which could potentially radically shift approaches towards control and elimination [118]. The development of anti-schistosome animal vaccines began in the 1970s with *S. mattheei*-irradiated larvae found to be as immunogenic in sheep as natural challenge [119], and with *S. bovis*-irradiated larvae found to be protective against natural challenge in cattle (higher growth rate, reduced worm burden and reduced egg output in vaccinated animals compared with controls) [117,120]. These initial attempts were followed in the 1980s by a cryopreserved radiation-attenuated schistosomula vaccine against *S. bovis* in sheep [121], as well as irradiated *S. japonicum* vaccines in mice, pigs, sheep, cattle and buffaloes of China [122,123]. More recent efforts have focused on the use of recombinant DNA vaccine technology for livestock­ (mostly testing three antigens: glutathione *S*-transferases, paramyosin and triose-phosphate isomerase) given its antibody- and cellular immunity-inducing properties, its cost, and logistical advantages of field deployment without relying on a cold-chain [117]. Despite the encouraging results of early live-attenuated *S. bovis* and *S. mattheei* livestock vaccine candidates, the lack of recently published studies [117] highlights a delay in progress and an unmet One Health research need.

### (e) One Health, environmental management and WASH

Implementation of water, sanitation and hygiene (WASH) measures (i.e. safe water supply, personal cleanliness and handwashing, access to facilities and services for the safe disposal of urine and faeces [5]), snail control and environmental management interventions, can reduce schistosome transmission and benefit the health of people, animals and ecosystems. However, the success of differing interventions will vary according to the local context. For instance, while chemical snail control with niclosamide molluscicide formulations resulted in a substantial reduction of snail populations in China, its use in Laos did not have a significant impact [26]. Moreover, molluscicide environmental contamination might preclude its use in other countries and hence more sustainable alternatives like environmental modification (e.g. concreting canals and/or vegetation removal in the snail habitat, land use change to avoid flood irrigation) and/or biological control (e.g. introduction of snail predators) could be explored in these contexts. In fact, the elimination of *S. japonicum* in Japan pre-dated praziquantel and relied on snail population reduction through environmental modification associated with rapid economic development, in combination with health education and animal reservoir control [26].

Key hygiene and sanitation deficiencies within Africa persist, including lack of sewage infrastructure (affecting two thirds of the continent's population) and no access to clean water (endured by almost half of the population in Africa) [83]. Robust long-term measures to tackle such challenges would greatly enhance the prospects of widespread and sustained elimination of schistosomiasis and other water-borne diseases. While a One Health approach has a role to play in improving this situation, it will ultimately require continued economic development, political commitment, and stability. It remains to be seen whether elimination of schistosomiasis can be achieved without such wholesale progress.

## 2. Conclusion

The importance of One Health to the control and elimination of NTDs has gained increased recognition through its integration into the WHO 2030 NTD roadmap [3] and companion documents [2,4,5]. The evolutionary history of schistosomes as multi-host parasites which continue to readily hybridize and adapt exemplifies the particular importance of One Health to the effectiveness and success of interventions targeting elimination. One Health approaches—which encompass the treatment and/or vaccination of livestock, environmental management, and snail control—will be integral to reducing the risk of zoonotic spillover and mitigating the risk of resurgence in human populations and improving the economic prospects and livelihoods of communities that depend on healthy livestock. Many challenges remain to the sustainable implementation of such approaches, but this should not detract from the importance of embracing One Health to combat schistosomiasis and other NTDs.

## Data Availability

This article has no additional data.
